# Exercise in the Park or Gym? The Physiological and Mental Responses of Obese People Walking in Different Settings at Different Speeds: A Parallel Group Randomized Trial

**DOI:** 10.3389/fpsyg.2021.728826

**Published:** 2021-10-22

**Authors:** Xinxin Wang, Quanfu Zhou, Mingjuan Zhang, Qinghai Zhang

**Affiliations:** ^1^Department of Landscape Architecture, College of Horticulture, Post-doctoral Research Station in Public Administration, Nanjing Agricultural University, Nanjing, China; ^2^Department of Sports, Nanjing Agricultural University, Nanjing, China; ^3^Department of Landscape Architecture, College of Horticulture, Nanjing Agricultural University, Nanjing, China; ^4^Key Laboratory of Landscaping, Ministry of Agriculture, Nanjing, China

**Keywords:** green exercise, physical activity, walking speed, health benefits, obesity

## Abstract

Evidence shows that physical activity has multiple health benefits for the body and mind of oneself, but little is known about the impacts of the setting and the intensity on exercise experience, especially for obese people. This study investigated the physiological and psychological effects of four walking conditions with different settings (park vs. gym) and intensity (slow vs. fast) on young obese adults. Subjects were 18–21 years old Chinese university students (*N* = 77), who were diagnosed as obese. They were randomly assigned to participate in one of the four activities in the field: slow walk in the park (2.8 km/h), fast walk in the park (5.5 km/h), slow walk in the gym, and fast walk in the gym. Physiological indices, including blood pressure and heart rate, were measured before and after the walk. Psychological responses were measured by the Symbol Digit Modalities Test, the mood states scale, and the semantic differential scale. This study of obese people aged 18–21 years confirmed the previous findings that exercising in natural environments better relieved stress and restored attentional level than indoor activities. The results suggested that the mood states of the participants and their environmental perceptions may be influenced by the walking conditions with different setting and speed. The findings can be used in planning and designing urban green spaces for promoting physical activity and making exercise plans for obese people.

## Introduction

Physical inactivity is a global public health problem. The World Health Organization (WHO) estimates that more than a quarter of people worldwide do not get a sufficient amount of exercise (Guthold et al., 2018), which raises the risk for obesity, diabetes, and other non-communicable diseases (Corrêa et al., 2016). Globally, 39% of adults (over 1.9 billion) aged 18 years and over were overweight in 2016, and among them, 13% (over 650 million) were found to be obese (World Health Organization, 2020). A government report by the National Health Commission of China revealed that 34.3 and 16.4% of Chinese adults were overweight and obese, respectively, suggesting a continuous increase in recent years (Fan, 2020). This trend raises concerns about physical inactivity and obesity, which have become major public health challenges (Wang et al., 2019).

Physical activity plays a critical role in maintaining a healthy body weight, as there is a vicious cycle of obesity and physical inactivity (Pietiläinen et al., 2008). Insufficient physical activity increases the risk of being overweight, and those who are already overweight or obese tend to participate in less physical activity than people with normal weight. Besides weight management, benefits of physical activity include bone and muscle strength promotion, a lower risk for cardiovascular disease, better sleep, reduced feelings of anxiety, and boosted endurance (Melzer et al., 2004; Warburton et al., 2006). It is commonly recommended that adults get at least 150 min of moderate aerobic activity or 75 min of vigorous aerobic activity a week (Piercy et al., 2018), but no specific guideline points out the impacts of exercise setting together with exercise intensity.

Studies have found that the features of an urban environment, such as passive transportation (Kjellström et al., 2007) and less open space (Ghimire et al., 2017), can contribute to the obesity epidemic. Green space may promote physical activity, and a number of studies have found that people living in areas with more green space undertake more physical activity (Shanahan et al., 2016). Evidence shows that access to green space is associated with a lower likelihood of obesity (Nielsen and Hansen, 2007; Xiao et al., 2020). It has been documented that exposure to nature is beneficial for children (Tillmann et al., 2018), teenagers (Tillmann et al., 2018), and older adults (Rodiek, 2002), but little is known about the potential benefits to obese people.

Green exercise refers to physical activity that takes place in outdoor environments with exposure to nature (Pretty et al., 2005). Green exercise has been found to have multiple positive effects, such as sustaining physical health (Lahart et al., 2019), reducing stress and anxiety (Gladwell et al., 2013), improving self-esteem and mood (Barton and Pretty, 2010), and promoting mental focus (Han, 2017). Although it is evident that exercise can improve health, several studies have found that green exercise can produce greater physical and mental health benefits than exercise in other settings (Thompson Coon et al., 2011; Mitchell, 2013). A field experiment conducted in Finland examined the psychological and physiological effects of visits to urban nature environments and found that, compared to a built-up city center, an urban park and urban woodland had positive effects on stress recovery (Tyrväinen et al., 2014). As physical activity in a gym is a viable alternative to outdoor exercise, comparing natural environments with gym sites could have important implications on physical activity interventions. Using photographs projected on a wall, a study from the United Kingdom tested the effects of exercising on a treadmill whilst exposed to images of nature, indicating that green exercise can better improve self-esteem than exercise only (Pretty et al., 2005).

Although these studies suggest the combined effects of green exercise, there is no differentiation of exercise intensity. The behavioral intensity of the activity, such as walking speed, is rarely mentioned in the previous literature, which could be of “fairly light” intensity, consisting of a slow walk no more than 4 km/h. On the other hand, research focused solely on exercise intensity found that the moderate intensity exercise in a gym increased positive well-being, but showed no change in the low–exercise intensity condition (Daley and Huffen, 2003). Based on the dual-mode theory, an empirical study found that people felt from pleasure to displeasure, as the intensity of exercise increased (Parfitt and Hughes, 2009). Since affective responses are affected by the activity level, the influence of the exercise intensity should be considered when analyzing the effects of green exercise (Ekkekakis et al., 2005). In addition, most participants in previous studies were young healthy adults or children. As obese individuals have different physical and behavioral traits from people with a normal weight, they tend to have little motivation to initiate and sustain physical activity. Green spaces might help support and maintain physical activity for obese people, thus special attention should be paid to the effects of green exercise on obesity prevention.

The context of sites where people take part in physical activity is the key factor to distinguish green exercise from indoor exercise. According to Attention Restoration Theory (Kaplan, 1995), environmental attributes and how people perceive them may affect the restorative experiences of individuals. Previous studies have shown that the environmental and spatial perceptions of people are positively correlated with their environmental preferences, and can influence their choices of behavior (van den Berg et al., 2003). Valuing the environmental perception of the participants on the exercise settings will help to understand their walking experience during the experiment.

This study investigated the psychophysiological responses of obese people with regard to the walking conditions with different exercise site and intensity: slow walk in the park, fast walk in the park, slow walk in the gym, and fast walk in the gym. It was hypothesized that exercise in natural environments would be more effective than exercise indoors in terms of stress recovery and attentional improvement for obese people. No hypothesis was formed regarding the impact of site and speed on mental affect and visual perception, but we expect that different walking conditions differ in changes of mood state and environmental perception.

## Methods

The Study methods are in accordance with the Consolidated Standards of Reporting Trials (CONSORT) guidelines for reporting randomized trials (Moher et al., 2010; Cuschieri, 2019). The trial was an open-label, single-center, parallel group study to evaluate the effects of walking setting and intensity on obese individuals. This study was carried out in accordance with the principles of the Declaration of Helsinki (World Medical Association, 2013). The study protocol was approved by the Experimental Animal Welfare and Ethics Committee of Nanjing Agricultural University, and all the participants provided written informed consent. This trial was sponsored by researchers from the Department of Sports and the Department of Landscape Architecture in Nanjing Agricultural University. One of the lead authors has established physical activity programs for obesity treatment as an elective course since 2015, open to college students who are obese. This trial was developed based on the content of the course.

### Study Design

Four types of behavioral interventions were used in the trial with different walking settings and walking speeds, including slow walk in the park, fast walk in the park, slow walk in the gym, and fast walk in the gym.

**Figure 1:**
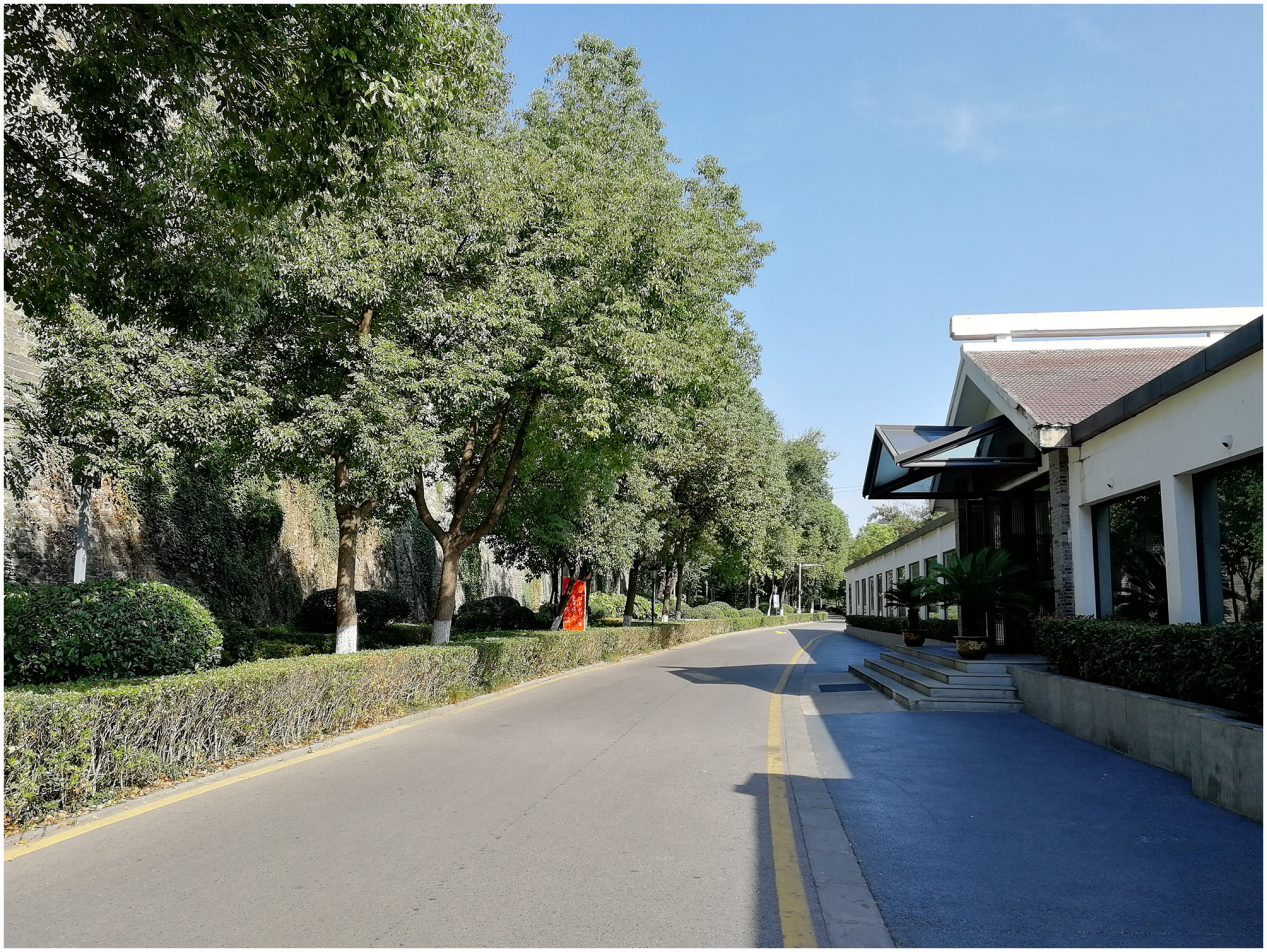
The park site for the experiment.

The urban park walking sessions took place in the Crescent Lake Park, which is located in the Qinhuai District, Nanjing, China (32°02′01.3″N, 118°49′40.5″E). The park was established in 1998 and covers an area of over 20 ha after two phases of construction. It is located next to the wall of the Ming Dynasty and surrounds the Crescent Lake in the center. The walking site chosen in the study was a section of the main walkway, which is flat with a gentle slope, connecting the southern and the northern gates of the park. As shown in Figure 1, the landscape quality of the walkway was generally ordinary: the width of the walkway was 4 m, with tall trees, shrubs, and hedges planted on both sides, and most plants were evergreen species, such as *Cinnamomum camphora, Magnolia grandiflora, Buxus sinica*, and *Pittosporum tobira*; two hotels were built on the east side of the walkway, taking a length of 150 m for the building interface, which partially blocked the view of the lake from the walkers. The length of the walkway was 800 m long, 1.6 km for a round-trip.

**Figure 2:**
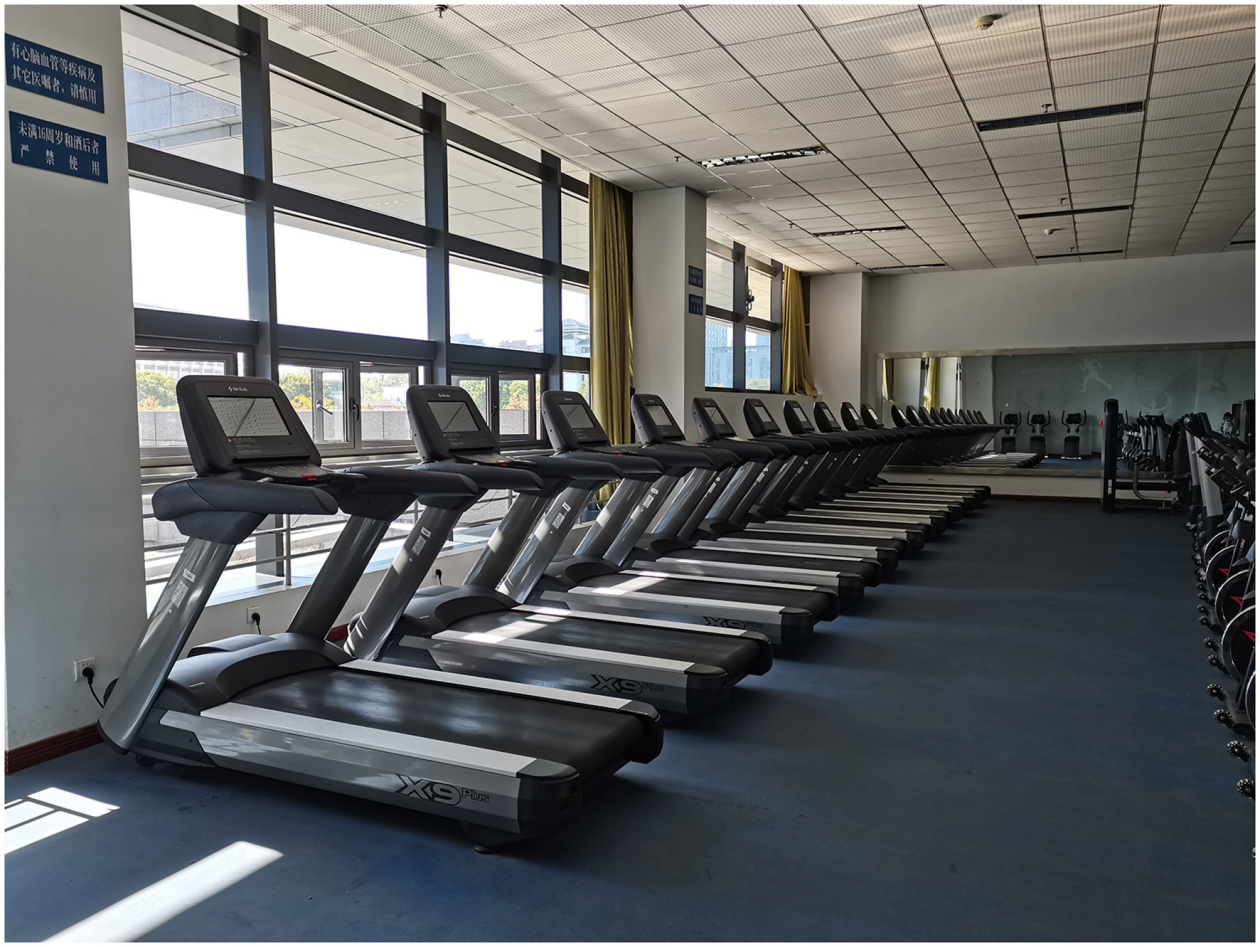
The gym site for the experiment.

The gym setting was located on the second floor of the sports center of the university. The gym faces south and covers an area of ~300 m^2^ with various fitness facilities. There are 10 treadmills in the gym, which can be used by multiple people at the same time (Figure 2). Through the windows, one can see the roof terrace on the first floor, the upper part of the street trees (*Cinnamomum camphora*), and several residential buildings in the distance. During the experiment, the treadmill was set at a 2% incline, to simulate the walkway conditions in the outdoors. The walking distance of the indoor group was the same as that of the outdoor group (1.6 km), and it was set on the treadmill.

The slow and fast walking speeds were decided based on the fitness guidelines by the US Department of Health Human Services (2018) and took into account the physical condition of the obese college students by the pretest procedure. According to the guide, the speed of a slow walk is 3.2 km/h or less, while the speed range for a fast walk is between 4.02 and 6.44 km/h. We then asked four adults with obesity (two men and two women) to walk slowly and walk fast as they liked. Their walking speeds were collected by a sports watch (Garmin Forerunner 235) wore on the left wrist. Although men tend to walk faster than women, the walking speeds of the participants were within the recommended range of the guide, and they agreed that a slow walk at 2.8 km/h and a fast walk at 5.5 km/h were appropriate.

During the experiment, the walking speed was controlled by research assistants for the outdoor group, and by treadmills for the indoor group. In the urban park setting, the walking group was led by one research assistant at the front of the queue, who kept moving at a constant speed using a metronome (Gudong, running apps) and a speedometer (Garmin Forerunner 235, sports watch). There was another research assistant at the end of the line, to help maintain the order. The treadmill has the function of speed setting, which makes it simple to control the speeds for the indoor group. It takes 34 min to finish the walking (1.6 km) at the slow speed (2.8 km/h), and 18 min at the fast speed (5.5 km/h). The interventions with different walking speeds were tested separately.

### The Participants

Eligible participants were 18–21 years old young adults (undergraduate students) who were obese with a body mass index (BMI) over 28 kg/m^2^ or a body fat percentage over 30%, according to the recommended criteria for the Chinese people (Zhou, 2002). It is worth mentioning that the conventional cut points for obesity (BMI ≥ 30 kg/m^2^ for obese) were adopted by most countries, the World Health Organization (2004) recommended lower BMI cut points for Asians (BMI ≥ 27.5 kg/m^2^ as obese), as the conventional cut points for overweight/obesity do not correspond to similar metabolic risk in all ethnic groups (Jih et al., 2014). Exclusion criteria included the diagnosis of cardiovascular disease, current use of any psychoactive drugs, or recent muscle/bone injury.

According to a survey conducted by one of the authors between 2013 and 2014, there were over 200 freshman and sophomore students in Nanjing Agricultural University with severe obesity (BMI ≥ 30 kg/m^2^). In the fall semester 2019–2020 when the trial took place, there were 112 students signed up for the obesity-specific fitness course (an elective course). Only obese students were allowed to take this course, and several rounds of measurements for height, weight, and body composition were conducted during the registration period and in the course. The health status of the obese students was also recorded as part of the study procedure, by asking them whether they have any diagnosed disease or mental disorder. We publicized this experiment with a contact group among the students enrolled in this course. After recruiting for a month, 80 obese college students agreed to participate in the experiment, and all of them met the eligibility criteria. As the participants were from the same fitness course, they had similar training levels and experiences in the walking activity.

**Figure 3:**
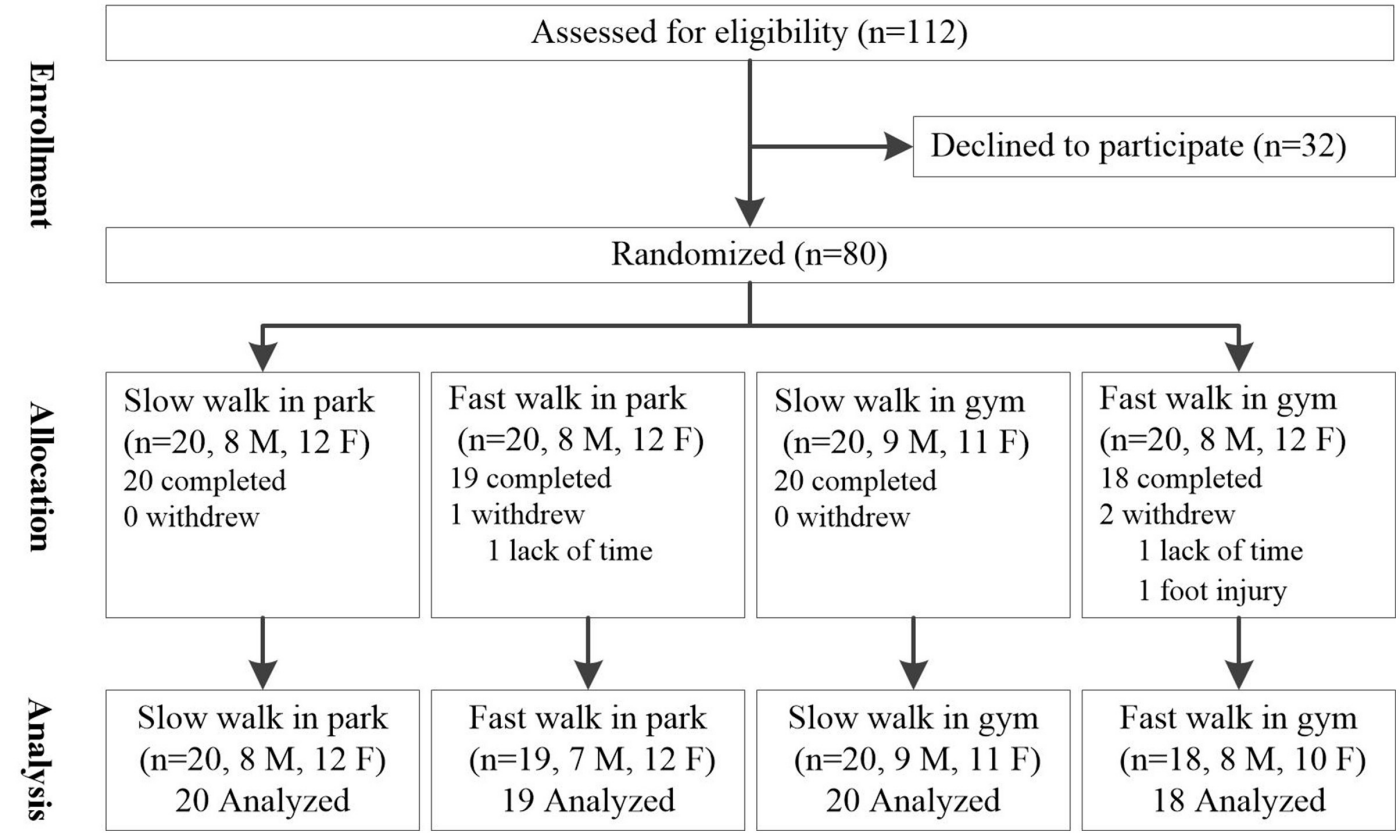
Flow diagram of the progress through the phases of a parallel randomized trial.

For allocation of the participants, two computer-generated randomization lists were prepared by one of the research assistants using the RAND function in Excel for men and women separately. Then the random numbers were sorted in ascending order, and were assigned to one of the four walking groups in sequence, initially in the ratio of 1:1:1:1. In this way, participants were randomly grouped, with similar ratio of men to women in each group (Figure 3). Three out of the 80 participants failed to participate in the experiment (one man and two women), as they had other assignments temporarily or were unwell on the day of the experiment. All withdrawals were unrelated to the walking intervention. In the end, 77 undergraduate students who were 18–21 years old (M = 19.1, S.D. = 0.7), from Nanjing Agricultural University, participated in the experiment, who were from a wide range of disciplines (12 different colleges). The mean BMI of the total sample was 31.7 kg/m^2^ (S.D. = 4.7), and their mean body fat percentage was 37.6% (S.D. = 5.2%). Table 1 shows the physical status of the subjects and their groups. There was no significant difference between the subjects in each group (*p* > 0.05).

**Table 1:** Means and standard deviations for the physical status of the subjects.

	**Exercise in park**		**Exercise in gym**	
	**Slow walk (*N* = 20)**	**Fast walk (*N* = 19)**	**Slow walk (*N* = 20)**	**Fast walk (*N* = 18)**
	**Mean (S.D.)**	**Mean (S.D.)**	**Mean (S.D.)**	**Mean (S.D.)**
Gender-%women	0.60	0.63	0.55	0.56
Age	19.26 (0.82)	19.33 (1.01)	19.01 (0.35)	18.87 (0.40)
Weight (kg)	96.42 (22.59)	83.69 (16.53)	93.58 (16.11)	92.95 (18.54)
Height (cm)	169.30 (8.02)	167.12 (8.91)	169.44 (8.27)	171.82 (8.94)
Body mass index (kg/m^2^)	33.32 (5.63)	29.78 (4.27)	32.41 (3.82)	31.25 (4.39)
Body fat percentage (%)	38.51 (4.71)	36.46 (5.74)	38.59 (5.07)	36.68 (5.29)

### Outcome Measures

#### Physiological Indices of Stress

Psychological stress can temporarily increase blood pressure and heart rate (HR), and relaxation could lower one's blood pressure and HR within the normal range. Exercise can also increase blood pressure and HR, as the sympathetic nervous system is activated during the exercise, and the physiological changes may remain after a few minutes of the recovery. For this study, a portable sphygmomanometer (Omren HEM-7211) was used to measure the systolic blood pressure (SP), diastolic pressure (DP), and HR of the subjects before and after the walking condition. It is recommended to rest for 5 min before beginning measurements (Williams et al., 2018).

#### Psychological Measures

Attentional level: Both the natural environment and physical activity have been found to increase cognitive control by restoring attention. In this study, the Symbol Digit Modalities Test (SDMT) (Smith, 1968, 1982) was used to evaluate the attentional level of the subjects. The reliability and the validity of the test have been confirmed in previous studies (Sheridan et al., 2006). The SDMT involves a simple substitution task and is easy to administer. The participant was given 90 s to pair specific numbers with given geometric figures. A reference example was shown at the top of the page, and participants were asked to write down the paired geometric figures in sequence in the table below. The score of the attentional level test was based on the number of correct answers.

Mood states: To detect the changing emotions of the subjects before and after the walking condition, we used the mood states scale “Befindlichkeitsskalen (BFS) (Abele-Brehm and Brehm, 1986).” The scale was developed to assess various aspects of subjective well-being, for both positive and negative mood states, including eight dimensions (activated, pleased, contemplative, calm, angry, agitated, depressed, and shiftless). The scale used in this experiment included 40 items, using a five-point rating system, from 1 = strongly disagree to 5 = strongly agree. The scale was originally developed in German, which was later introduced to China in 1994 and translated into Chinese. The reliability and validity of the Chinese version of the BFS have been tested in previous studies (Si and Huang, 1997).

Environmental perception: The semantic differential (SD) scale was used to evaluate the environmental quality of the different exercise sites, and to measure the potential impact of the walking speed on environmental perception. Fifteen pairs of bipolar adjectives were extracted from the literature related to environmental assessment (e.g., artificial-natural, closed-open) (Natori and Chenoweth, 2008; Song et al., 2017). After completing the walking exercise, the participants were asked to use the 7-point bipolar rating scale coded from −3 to +3 to indicate their attitudes to the sites.

### The Procedure

The experiment was conducted from August to December 2019. The day before the formal experiment, the research assistants sent mobile messages to the participants for the next day, notifying them the gathering time and place, and reminded them do not smoke, do not drink coffee or alcohol before the experiment, and wear workout clothes. We arranged 8–10 participants for each group, and they were accompanied by five trained research assistants.

When the participants arrived for their appointment, a research assistant provided them with a general description of the experimental procedures, and obtained the informed consent from the participants. The participants were asked to turn off their phones and headphones before the experiment, to reduce the possible influence of other factors. After the subjects filled in the basic information, they sat and rested for at least 5 min before baseline measurements were taken for blood pressure and HR. Afterward, the research assistants guided the participants to do the SDMT to test their attentional level, and then to complete the BFS scale to measure their mood states at the moment. While they conducted the walking exercise, the participants were asked to experience the surroundings, and they were not allowed to communicate with each other during the walk. The walking distance for all groups was 1.6 km. When the walking stage was finished, the participants did the SDMT and BFS scale again. After sitting and resting for 5 min, the blood pressure and HR of the participants were measured. They also did the SD scale to indicate their environmental perceptions for the walking site they just experienced.

In order to start the trial at the same time, the participants who were assigned a walk in the park gathered near the north gate of the university at 1:50 pm. To reduce the influences of physical activity before the experiment, the participants and the research assistants were delivered to the park site by car, a trip that took ~10 min. For walking groups in the gym, the participants arrived at the sports center of the university at 2:00 pm. The participants in the park setting were walking in line with a distance of about 2 m, while subjects in the gym were walking in row on the treadmill.

### Data Analysis

As the physiopsychological indices of the subjects were measured at two time points (before and after the walking session), and measures across time were probably not independent, multilevel modeling was used to investigate the impacts of site and speed on walking experience. The repeated measurements (level 1) were nested within the individual (level 2). In order to examine the effects of nesting on the results, the unconditional means models were fitted to estimate the intraclass correlation coefficient (ICC). Then several conditional growth models were fitted to determine how much a walking session will change the physiological and psychological indices of the subjects. SP, DP, HR, and mood states were used in the models as the outcome variables, respectively. The time points, the site types, the speed levels, and the interactions were included in the models as the fixed effects, and the repeated observations nested within individuals were treated as the random effect. Multilevel modeling package “nlme” in R was used to analyze the data (Pinheiro et al., 2021). The SD scale was treated as an ordinal scale (Agresti, 2010), and the Mann–Whitney *U* tests were used to analyze the differences in environmental perception between the two different exercise settings at different walking speeds. The level of significance was set at *p* < 0.05. The statistical analysis was completed in R 4.1.1.

## Results

### Tests for the Effects of Different Sites and Walking Speeds on Physiological Indices

**Figure 4:**
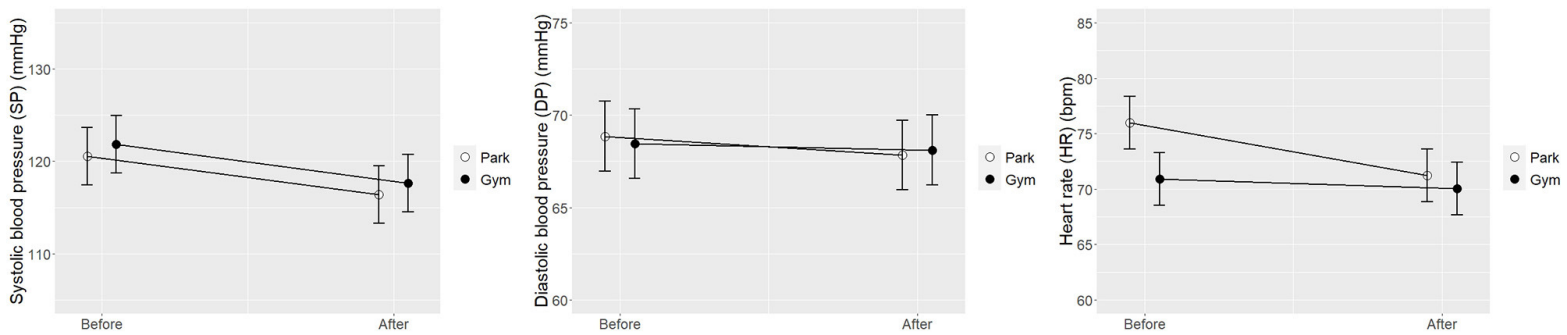
Interaction plots of estimated marginal means with SEM error bars between the time points and walking sites.

**Figure 5:**
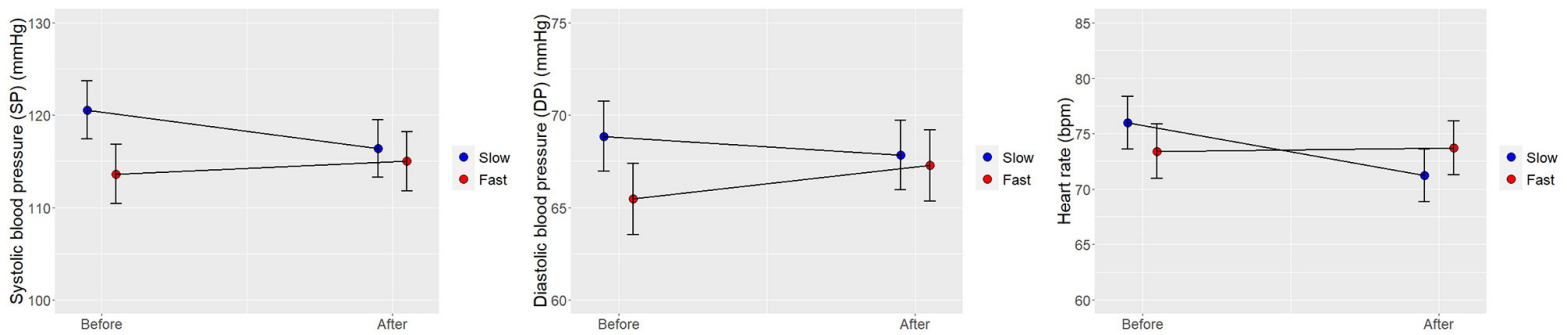
Interaction plots of estimated marginal means with SEM error bars between the time points and walking speeds.

The unconditional means models were fitted to see if the nested variable at level 2 significantly affects the intercept of the outcome variable at level 1. The ICCs for the three models on the physiological indices ranged from 0.76 to 0.86, indicating a great impact of clustering and therefore the necessity for multilevel modeling strategy (Musca et al., 2011). As presented in Table 2; Figure 4, there was a small reduction in the SP for both park and gym groups, but no significant differences in changes of SP and DP values between the setting groups were found. Compared with walking in the gym, walking in the park tended to be more effective in reducing the HR of the participants over a period of time (*B* = 3.85, *t* = 2.76, 95% CI: 1.13–6.57), which may be due to the stress recovery effects of the natural environment. As shown in Table 2; Figure 5, the changes in physiological indices of the subjects were impacted by the speed of walking. Compared with walking at the slow speed, fast walking was more likely to raise the SP (*B* = 5.55, *t* = 3.42, 95% CI: 2.39–8.71), DP (*B* = 2.82, *t* = 2.08, 95% CI: 0.18–5.46), and HR (*B* = 5.01, *t* = 3.59, 95% CI: 2.29–7.73) of the participants over a period of time. According to the interaction plots between time and speed (Figure 5), the marginal means of the three physiological indices were slightly reduced by the slow walking condition, which indicates slow walking may be better at maintaining blood pressure and HR within normal limits.

**Table 2:** Multilevel modeling results for the effects of walking conditions on physiological indices, including systolic blood pressure, diastolic blood pressure, and heart rate.

	**Model 1-SP**		**Model 2-DP**		**Model 3-HR**	
	**B**	**SE**	**B**	**SE**	**B**	**SE**
**Fixed effects**						
(Intercept)	120.56^***^	3.19	68.86^***^	1.93	75.96^***^	2.43
Time^1^	−4.17^**^	1.39	−1.01	1.16	−4.72^***^	1.19
Site^2^	1.28	4.48	−0.41	2.69	−5.07	3.41
Speed^3^	−6.93	4.54	−3.39	2.72	−2.55	3.45
Time: Site	−0.07	1.62	0.68	1.36	3.85^**^	1.40
Time: Speed	5.55^**^	1.62	2.82^*^	1.36	5.01^***^	1.40
Speed: Site	6.38	6.36	4.62	3.75	6.24	4.82
**Random effects**						
Individual	13.17 (4.92)		7.49 (4.11)		9.87 (4.23)	

1 *The variable “time” was coded as 0 (before the walking condition) and 1 (after the walking condition)*.

2 *The variable “site” was coded as 0 (park setting) and 1 (gym setting)*.

3 *The variable “speed” was coded as 0 (slow speed, 2.8 km/h) and 1 (fast speed, 5.5 km/h)*.

* *p < 0.05*,

** *p < 0.001*,

*** *p < 0.001*.

### Tests for the State of Attention Before and After Walking

The changes in attentional level of the participants before and after walking were measured with the Symbol Digit Modalities Test (SDMT). The ICC for the unconditional means model of attentional level was 0.71, which indicates the need to employ multilevel modeling. As shown in Table 3, the model showed no interaction effects of exercise sites and walking speeds on the changes in attentional level. The state of attention of the subjects was generally increased over the period of walking (*B* = 7.84, *t* = 6.55, 95% CI: 5.51–10.18). Walking in the park was more likely to increase the subjects' attentional level than walking in the gym over the period (*B* = −3.79, *t* = −2.70, 95% CI: −6.52 to −1.06), which may suggest that the park setting was more restorative than the gym setting.

**Table 3:** Multilevel modeling results for the effects of walking conditions on the attentional level.

	**Model 4-Attentional level**				
	**B**	**SE**	***t*-Value**	**95% CI**	
				**Lower**	**Upper**
**Fixed effects**					
(Intercept)	69.43^***^	2.21	31.44	65.13	73.73
Time^1^	7.84^***^	1.20	6.55	5.51	10.18
Site^2^	10.49^**^	3.09	3.40	4.48	16.50
Speed^3^	5.54	3.12	1.77	−0.54	11.63
Time: Site	−3.79^**^	1.40	−2.70	−6.52	−1.06
Time: Speed	−1.84	1.40	−1.31	−4.57	0.89
Speed: Site	−6.60	4.34	−1.52	−15.05	1.84
**Random effects**					
Individual	8.79 (4.25)				

1 *The variable “time” was coded as 0 (before the walking condition) and 1 (after the walking condition)*.

2 *The variable “site” was coded as 0 (park setting) and 1 (gym setting)*.

3 *The variable “speed” was coded as 0 (slow speed, 2.8 km/h) and 1 (fast speed, 5.5 km/h)*.

** *p < 0.01*,

*** *p < 0.001*.

### Tests for the Mood States Before and After Walking

The mood survey scale “Befindlichkeitsskalen (BFS)” was used to measure changes in positive and negative mood states before and after the walking exercise. The ICCs for the models on positive mood states ranged from 0.66 to 0.85, and the ICCs for the models on negative mood states ranged from 0.32 to 0.46. The results of the multilevel models are shown in Tables 4, 5. The data indicated that walking was likely to increase the feelings of contemplation in subjects (*B* = 0.25, *t* = 2.42, 95% CI: 0.050.45) and reduced their feelings of agitation (*B* = −0.34, *t* = −3.84, 95% CI: −0.52 to −0.17) over a period of time. The improvement of emotions in different dimensions might be associated with the walking sites and the walking speeds. It seemed that walking in the park was more likely to promote feelings of calmness in the subject than walking in the gym (*B* = −0.31, *t* = −2.13, 95% CI: −0.59 to −0.03), and walking at a slow speed was more likely to increase the feelings of contemplation than walking at a fast speed (*B* = −0.34, *t* = −2.78, 95% CI: −0.58 to −0.10).

**Table 4:** Multilevel modeling results for the effects of walking conditions on the positive moods.

	**M5-activated**		**M6-pleased**		**M7-contemplation**		**M8-calm**	
	**B**	**SE**	**B**	**SE**	**B**	**SE**	**B**	**SE**
**Fixed effects**								
(Intercept)	2.59^***^	0.20	3.06^***^	0.20	1.88^***^	0.15	2.79^***^	0.16
Time^1^	−0.11	0.14	−0.07	0.14	0.25^*^	0.10	0.23	0.12
Site^2^	−0.33	0.27	−0.55	0.28	−0.07	0.20	−0.13	0.23
Speed^3^	−0.21	0.28	−0.26	0.29	−0.06	0.20	−0.27	0.23
Time: Site	0.11	0.17	0.04	0.16	−0.13	0.12	−0.31^*^	0.14
Time: Speed	0.34^*^	0.17	0.30	0.16	−0.34^**^	0.12	−0.05	0.14
Speed: Site	0.50	0.38	0.66	0.39	0.21	0.28	0.55	0.31
**Random effects**								
Individual	0.72 (0.50)		0.75 (0.50)		0.54 (0.37)		0.59 (0.43)	

1 *The variable “time” was coded as 0 (before the walking condition) and 1 (after the walking condition)*.

2 *The variable “site” was coded as 0 (park setting) and 1 (gym setting)*.

3 *The variable “speed” was coded as 0 (slow speed, 2.8 km/h) and 1 (fast speed, 5.5 km/h)*.

* *p < 0.05*,

** *p < 0.001*,

*** *p < 0.001*.

**Table 5:** Multilevel modeling results for the effects of walking conditions on the negative moods^4^.

	**M9-angry**		**M10-agitated**		**M11-depressed**		**M12-shiftless**	
	**B**	**SE**	**B**	**SE**	**B**	**SE**	**B**	**SE**
**Fixed effects**								
(Intercept)	1.29^***^	0.08	1.57^***^	0.09	1.49	0.10	1.62^***^	0.15
Time^1^	−0.05	0.09	−0.34^***^	0.09	−0.10	0.10	0.13	0.14
Site^2^	0.03	0.11	0.04	0.13	−0.07	0.14	0.35	0.21
Speed^3^	−0.14	0.11	−0.12	0.13	−0.22	0.14	−0.05	0.21
Time: Setting	0.05	0.10	0.07	0.10	0.01	0.12	−0.28	0.17
Time: Speed	−0.02	0.10	0.14	0.10	−0.05	0.12	−0.31	0.17
Speed: Setting	0.05	0.14	−0.03	0.17	0.14	0.18	0.03	0.28
**Random effects**								
Individual	0.21(0.32)		0.29(0.32)		0.29(0.35)		0.48(0.50)	

1 *The variable “time” was coded as 0 (before the walking condition) and 1 (after the walking condition)*.

2 *The variable “site” was coded as 0 (park setting) and 1 (gym setting)*.

3 *The variable “speed” was coded as 0 (slow speed, 2.8 km/h) and 1 (fast speed, 5.5 km/h)*.

4 *Negative values in this table indicate a decrease in negative mood*.

*** *p < 0.001*.

### Environmental Perceptions for the Different Sites

**Figure 6:**
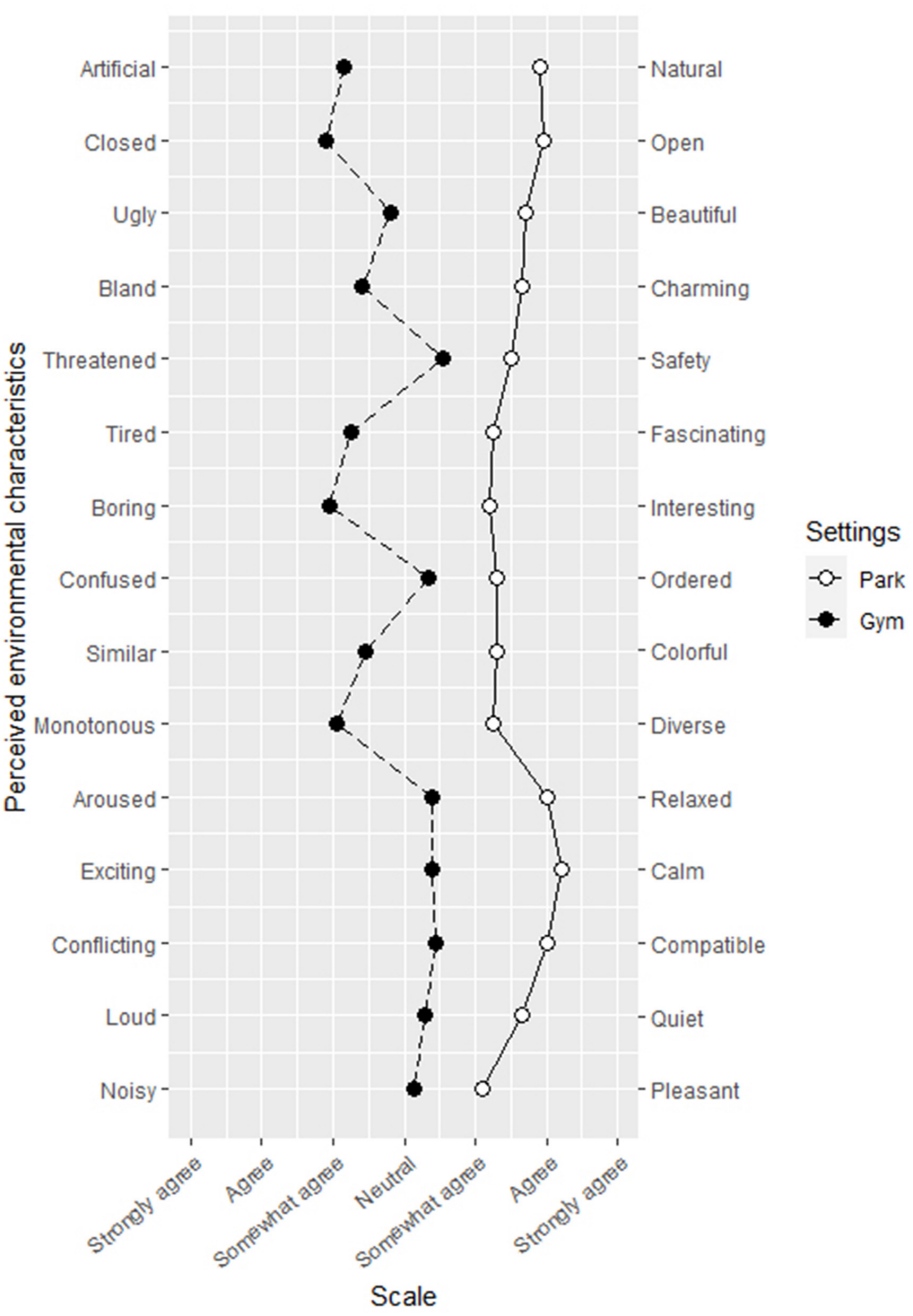
Measurements for the perception of park and gym settings while walking slowly using semantic differential scales.

**Figure 7:**
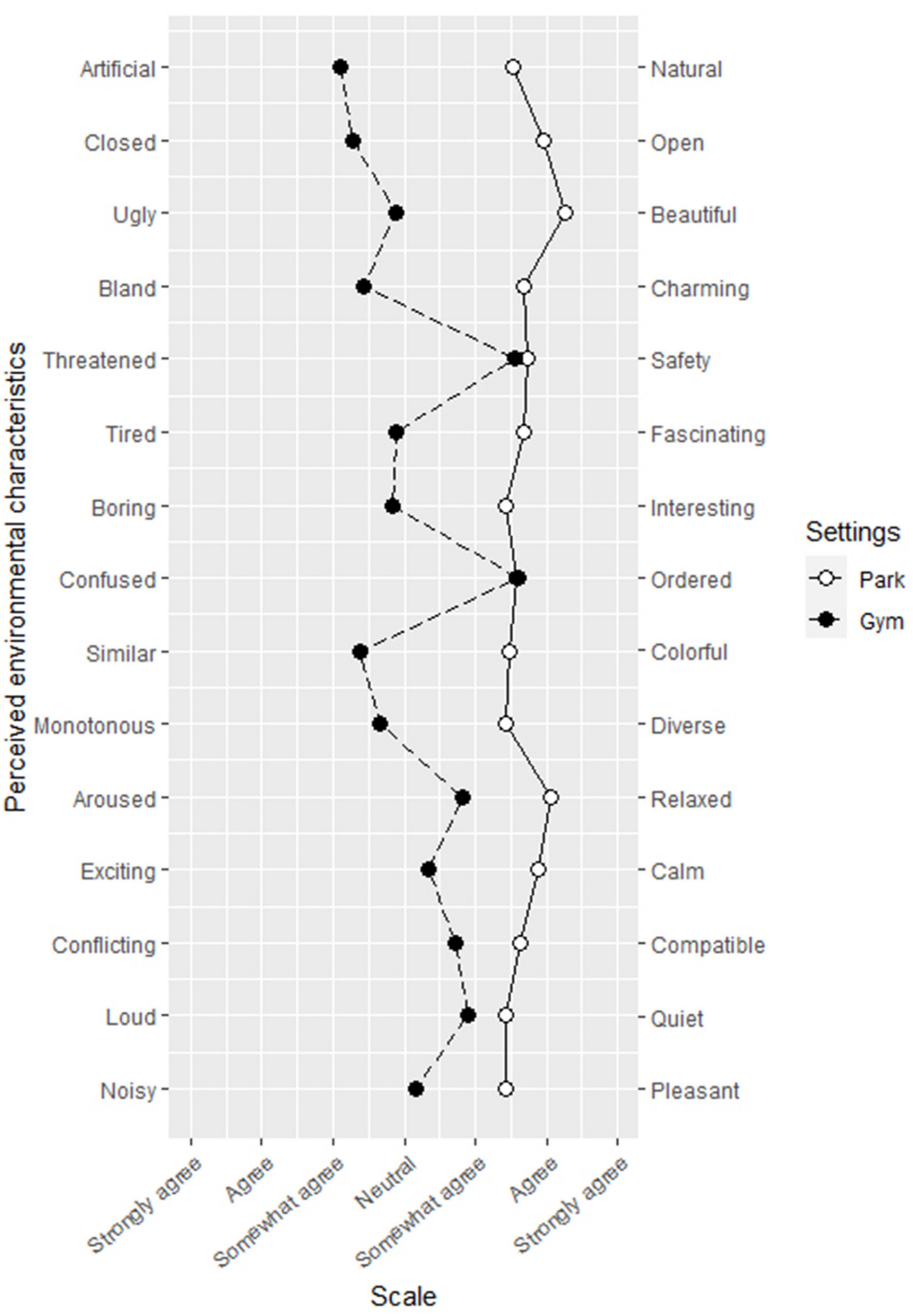
Measurements for the perception of park and gym settings while walking fast using semantic differential scales.

The internal consistency of the SD scale used for analyzing environmental perceptions was acceptable, with Cronbach's α ranging from 0.77 to 0.91. As data were measured on an ordinal scale, the Mann–Whitney *U* tests were used to compare the differences in environmental perceptions between the groups. As presented in Table 6; Figure 6, at the slow walking speed (2.8 km/h), participants tended to endorse higher scores for the park site than the gym site; significant differences were found for the items of natural, open, beautiful, charming, fascinating, interesting, diverse, relaxed, calm (*p* < 0.001), colorful (*p* = 0.001), compatible (*p* = 0.002), quiet (*p* = 0.009), and pleasant (*p* = 0.023), but no significant difference was found between the two sites for the items of safety (*U* = 136, *p* = 0.086) and ordered (*U* = 140, *p* = 0.108). As shown in Table 7; Figure 7, at the fast walking speed (5.5 km/h), again the items were higher rated for the park site. No significant difference was found between the two sites for the items of safe (*U* = 165, *p* = 0.845), ordered (*U* = 153, *p* = 0.599), and quiet (*U* = 159, *p* = 0.730). For the item of ordered, the mean rank for the gym setting (20.0) was slightly higher than that of the park setting (18.1). Based on the *U* values for each item, the environmental perceptions for the two sites differed less for the fast walking groups compared with the slow walking groups, except for the items natural, colorful, and pleasant. The results also suggest that the subjects, who completed the same distance of walking at different walking speeds, may have different environmental perceptions for the same exercise setting. For example, for the gym exercisers, the fast walkers rated higher on the items interesting (*U* = 108, *p* = 0.035) and ordered (*U* = 112, *p* = 0.048) than the slow walkers.

**Table 6:** Comparison of environmental perceptions for different settings while walking slowly.

	**Exercise in park (*N* = 20)**	**Exercise in gym (*N* = 20)**	***U***	***p***
	**Mean rank**	**Mean rank**		
Artificial-Natural	26.73	14.28	76	<0.001^***^
Closed-Open	29.53	11.48	20	<0.001^***^
Ugly-Beautiful	29.45	11.55	21	<0.001^***^
Bland-Charming	29.55	11.45	19	<0.001^***^
Threatened-Safe	23.70	17.30	136	0.086
Tired-Fascinating	29.03	11.98	30	<0.001^***^
Boring-Interesting	28.40	12.60	42	<0.001^***^
Confused-Ordered	23.50	17.50	140	0.108
Similar-Colorful	26.70	14.30	76	0.001^**^
Monotonous-Diverse	28.00	13.00	50	<0.001^***^
Aroused-Relaxed	27.43	13.58	62	<0.001^***^
Exciting-Calm	27.40	13.60	62	<0.001^***^
Conflicting-Compatible	26.18	14.83	87	0.002^**^
Loud-Quiet	25.30	15.70	104	0.009^**^
Noisy-Pleasant	24.70	16.30	116	0.023^*^

* *p < 0.05*,

** *p < 0.01*,

*** *p < 0.001*.

**Table 7:** Comparison of environmental perceptions for different settings while walking fast.

	**Exercise in park (*N* = 19)**	**Exercise in gym (*N* = 18)**	***U***	***p***
	**Mean rank**	**Mean rank**		
Artificial-Natural	24.11	13.61	74	0.003^**^
Closed-Open	25.89	11.72	40	<0.001^***^
Ugly-Beautiful	25.89	11.72	40	<0.001^***^
Bland-Charming	24.79	12.89	61	0.001^**^
Threatened-Safe	18.66	19.36	165	0.845
Tired-Fascinating	25.21	12.44	53	<0.001^***^
Boring-Interesting	25.08	12.58	56	<0.001^***^
Confused-Ordered	18.05	20.00	153	0.599
Similar-Colorful	24.50	13.19	67	0.001^**^
Monotonous-Diverse	23.53	14.22	85	0.008^**^
Aroused-Relaxed	23.53	14.22	85	0.008^**^
Exciting-Calm	24.63	13.06	64	0.001^**^
Conflicting-Compatible	23.16	14.61	92	0.016^*^
Loud-Quiet	19.63	18.33	159	0.730
Noisy-Pleasant	23.32	14.44	89	0.012^*^

* *p < 0.05*,

** *p < 0.01*,

*** *p < 0.001*.

## Discussion

### Walking at Different Sites and Different Speeds

This study investigated the effects of the different sites and the walking speeds on stress recovery, attention restoration, changes in mood states, and environmental perceptions. In a sample of obese young adults, the findings of this experiment suggest that exercising in a gym may have several positive effects, while exercising in a park could offer greater health benefits, which generally agrees with previous research on other populations (Pretty et al., 2005; Wooller et al., 2018). The results also suggest that the walking speed might have an impact on the walking experience of the subjects.

The results from physiological measures indicated that both speed and site influenced the blood pressure and HR of the subjects during the walking condition. Blood pressure and HR can be temporarily increased by exercise, and may remain high for a few minutes after finishing the walking. The increase in blood pressure and HR caused by exercise can be partially offset by the natural environment. The park site was likely more effective in stress reduction than the gym site, although the park used in the experiment was of ordinary quality.

Exercising the body can help improve the attentional level, and a green exercise environment may have additional benefits on mental restoration. The results from the SDMT test showed that the average attentional level of the subjects conducting the walking condition were all improved. The improved number of correct answers for the park walkers was more than that of the gym walkers. It is suggested that the park site was more restorative than the gym site. This supports previous research, suggesting that contact with nature-based components can lead to enhanced attentional restoration (Kaplan, 1978).

Comparing the mood states of the subjects before and after the walk, this study found that walking condition may slightly promote certain dimensions in positive emotions (e.g., contemplation) and decrease certain negative emotions (e.g., agitation). People tend to gain different mental benefits from exercise at different sites and at different intensities. This supports previous research showing that exercise of moderate intensity and exposure to nature induce a greater improvement of the mood states than low-intensity exercise in an unnatural environment (Daley and Huffen, 2003; Thompson Coon et al., 2011).

The results from the SD scale showed that the park site was associated with more positive environmental perceptions, regardless of intensity. The park site was rated higher in fascinating, interesting, relaxed, compatible, and pleasant, indicating that the participants seem to perceive the park as being more restorative. In this experiment, the gym site was rated higher by the fast walkers than the slow walkers on the items of interesting and ordered (*p* < 0.05), and the same went for the park walkers, although the difference did not reach significance. It is likely that the walking speed or the duration of the walking session may have an influence on the environmental sensitivity of the individuals. As factors such as personality traits and some underlying neurobiological mechanisms may also affect the sensitivity of perception of people (Greven et al., 2019), more research is needed to determine the relationship.

### Green Exercise for Obese People

Compared with healthy adults, obese people need to do more exercise to avoid regaining weight. However, individuals who are overweight or obese are often reluctant to participate in physical activity because the same amount of exercise for an obese individual will be much harder than for a healthy-weight person. Exercise in natural settings may feel more enjoyable, making it easier for people to stick to a workout routine. This suggests that “green” elements might actually encourage physical activity, a result that has been supported by existing research (Lacharité-Lemieux et al., 2015; Han, 2017; Loureiro and Veloso, 2017). For individuals with obesity, green exercise can benefit them more for at least two reasons. First, when conducting physical activity of the same intensity, green exercise may allow individuals to maintain a normal HR and blood pressure more easily than indoor exercise. People who are obese are at an increased risk for cardiovascular diseases (Lavie et al., 2018). Engaging in a higher level of physical activity may cause their HR and blood pressure to rise to levels that may be unsafe (Eijsvogels et al., 2016). In this study, we found walking in the natural environment, even a park of average quality, can lower HR and blood pressure, and partially offset the raised blood pressure caused by exercise. Second, green exercise has additional mental health benefits than indoor activity, which is important for obese people. Obesity is closely related to mental health issues, as excess stress can cause weight gain, and obesity is associated with increased risk of depression (McElroy et al., 2004). Engaging in green exercise may help obese people cope effectively with these issues, since it has been associated with improved stress recovery, enhanced attentional restoration, and a reduction in negative emotions. It is worth mentioning that no negative effects of exercising in the gym were found in the study, and gym exercise may also have some positive effects in enhancing attentional level and positive emotions.

### Strengths and Limitations

This study compared the psychophysiological responses and environmental perceptions of obese college students walking in different settings and at different speeds. The tested walking sites are two types of space frequently used by people for daily physical activity. This study may help obese exercisers understand the health benefits of walking in the park and the gym.

Because of the limited sample size, this study may not have the power to reveal all the effects, possibly resulting in a type II error. Also due to the limited sample size, when comparing the park site with the gym site, only one park of ordinary quality was investigated. Future research could explore the influence of walking activity across more types of urban green spaces. The samples for this experiment relied on an obese population among the Chinese university students, who were fairly homogeneous in terms of age, educational background, and daily lifestyles, therefore future studies should include people with different occupations and from different countries, in order to generalize the findings to a larger population. In addition, the subjects were not subdivided into groups based on their demographic characteristics and behavioral preferences. Because walking experience of people may be affected by their motivations to exercise (Manfredo et al., 1996), future studies could take personal intrinsic factors into consideration, to further detect the activity types and the environmental preferences of people with different characteristics.

## Conclusions

Urban parks are vital for our daily health and well-being, providing essential opportunities to be physically active. This study found that the park setting tended to be more effective in relieving stress in obese people and promoting their attention than the indoor gym, although the park tested was of average quality. The findings imply that cities need to provide enough green spaces for people to increase fitness and reduce obesity. Exercise intensity (e.g., walking speed) may potentially affect the mood states and environmental perception of people, suggesting individuals should take their mental needs into account when developing an activity plan. The findings can also help landscape architects design outdoor exercise settings based on the behavioral characteristics of the users. Walkways can be designed to satisfy the participants with various physical conditions and activity needs. For example, specialized trails with softer surfaces and supportive facilities may be more appealing to obese people, thus will help increase their amount of exercise in the outdoors. Besides, establishing walkways suitable for different exercise intensities, such as routes for leisure walking, running, and hiking, can avoid conflicts among the user groups and improve the safety of the sites. By designing green spaces of good quality, more opportunities for outdoor recreation can be provided, which will be helpful for public health.

## Data Availability Statement

The raw data supporting the conclusions of this article will be made available by the authors, without undue reservation.

## Ethics Statement

The studies involving human participants were reviewed and approved by the Experimental Animal Welfare and Ethics Committee of Nanjing Agricultural University. The patients/participants provided their written informed consent to participate in this study.

## Author Contributions

XW: conceptualization. XW and QZha: funding acquisition. XW and QZho: investigation. XW and MZ: statistics. QZha: supervision. XW and MZ: writing—original draft. All authors contributed to the article and approved the submitted version.

## Funding

This research was funded by the National Natural Science Foundation of China (Nos. 51808295 and 51708343), the Fundamental Research Funds for the Central Universities (No. KJQN201929), and the Forestry Science and Technology Innovation and Promotion Project in Jiangsu Province (No. LYKJ[2020]16).

## Conflict of Interest

The authors declare that the research was conducted in the absence of any commercial or financial relationships that could be construed as a potential conflict of interest.

## Publisher's Note

All claims expressed in this article are solely those of the authors and do not necessarily represent those of their affiliated organizations, or those of the publisher, the editors and the reviewers. Any product that may be evaluated in this article, or claim that may be made by its manufacturer, is not guaranteed or endorsed by the publisher.
